# Evaluation of corrective actions of feedback from clinicians on Clinical Laboratory Services at Bamenda Regional Hospital Laboratory, Cameroon

**DOI:** 10.4102/ajlm.v9i1.843

**Published:** 2020-03-23

**Authors:** Victor N. Fondoh, Charles N. Awasom, Rebecca Enow-Tanjong, Richard M. Fondoh, Patrick Njukeng, Judith Shang, Julianna Ndasi, Moses Samje, Claris N. Muluh, Thompson N. Kinge

**Affiliations:** 1 Bamenda Regional Hospital Laboratory, Regional Hospital Bamenda, Cameroon; 2 Faculty of Health and Medical Sciences, Catholic University of Bamenda, Bamenda, Cameroon; 3 North-West Regional Fund for Health Promotion, Bamenda, Cameroon; 4 Global Health Systems Solutions, Limbe, Cameroon; 5 Center for Disease Control and Prevention, Yaounde, Cameroon; 6 Faculty of Health Sciences, University of Bamenda, Bamenda, Cameroon; 7 Administration, Regional Hospital Bamenda, Bamenda, Cameroon

**Keywords:** evaluation, feedback, corrective actions, clinician satisfaction, clinical laboratory services

## Abstract

**Background:**

Customers’ satisfaction is imperative for success. Clinical laboratories continuously strive to attain very high levels of customer satisfaction to serve their clients and maintain accreditation. The concept of customer satisfaction has not yet been asserted in most clinical laboratories in Cameroon.

**Objectives:**

Our objectives were to assess the satisfaction of clinicians with the laboratory services at the Bamenda Regional Hospital Laboratory, identify important challenges, corrective actions implemented and changes in satisfaction.

**Methods:**

This retrospective study reviewed secondary data from clinician satisfaction survey records from March 2017 and November 2017. Challenges and implemented corrective actions were identified for assessed statements of dissatisfaction (dissatisfaction rates ≥ 20%) on the March 2017 survey. Satisfaction rates in March 2017 and November 2017 were compared.

**Results:**

High levels of dissatisfaction were observed for general satisfaction, waiting time, communication, duty consciousness, specimen collection and approach on the March 2017 survey. The main challenges identified were: lack of respect for the expected length of the waiting time, poor attitude, inadequate information, staff shortage and inadequate supervision. Statistically significant reductions in rates of dissatisfaction were observed for general satisfaction, waiting time, communication, response to emergencies, issuing of results, specimen collection, approach and duty consciousness.

**Conclusion:**

Waiting time is a major cause of clinician dissatisfaction with laboratory services. The identification of clinicians’ challenges and the effective implementation of corrective actions contribute to improvements in clinician satisfaction.

## Introduction

For every business to succeed, customer satisfaction is imperative. The satisfaction of customers at health institutions such as clinical laboratories, which includes clients (patients) and clinicians (doctors, nurses), is evaluated by using customer satisfaction surveys to continuously improve services.^1,2,3,4^ Besides the continuous striving to attain very high levels of customer satisfaction, clinical laboratories must maintain their customers and competence in compliance with the international standards and their accreditation. This is done through the effective implementation of the International Organization for Standardization (ISO) 15189 and ISO 17025 Standards.^4,5,6^ Feedback from laboratory customers is a compulsory quality indicator in the preparation of the balanced scorecard for monitoring the effectiveness of the quality management system.^4,6^ One of the tools used to assess feedback is the clinician satisfaction survey.

Many previous studies have assessed clinicians’ satisfaction with clinical laboratories in Europe, America^7,8^ and Africa.^9^ Findings from these studies reveal that most clinicians were satisfied with the cleanliness of the environment, quality of the results, presentation of the results, sample collection and handling of samples, whereas others were dissatisfied with the waiting times and attitude of the staff.^8,9,10,11^ Most of these studies did not identify the corrective actions implemented or document changes in satisfaction. Although clinician satisfaction surveys are conducted for clinical laboratories in Cameroon that are enrolled in the Stepwise Improvement Process Towards Accreditation,^12,13,14^ there is little published literature on the topic.

The Regional Hospital Bamenda is a public hospital that uses feedback from customer satisfaction surveys from patients to identify departments in the hospital that they are dissatisfied with. Feedback from the surveys are presented to the clinicians and heads of the departments during the weekly hospital coordination meetings. However, corrective actions are not evaluated, and the questionnaires used are not standard. Besides, apart from the laboratory services, the concept of a ‘clinician survey’ has not yet been established by other departments of the hospital. The Bamenda Regional Hospital Laboratory is a business centre in the Regional Hospital Bamenda. It is a newly accredited clinical laboratory^15^ that routinely makes use of customer satisfaction surveys as a tool to assess customer feedback. Surveys are done twice a year and corrective actions implemented in order to continuously improve on the quality of service delivery. Our objectives were to assess clinicians’ satisfaction with the laboratory service, identify challenges, corrective actions implemented and document changes in satisfaction.

## Methods

### Ethical considerations

Ethical approval to carry out this study was obtained from the Institutional Review Board (IRB) of the Bamenda Regional Hospital (IRB reference number: 18/APP/RDPH/RHB/IRB). Consent to participate in the survey was obtained verbally (by agreeing to fill out the questionnaire).

### Study area

The study was conducted at Bamenda Regional Hospital Laboratory (BRHL), which is a department at the Regional Hospital Bamenda. The Regional Hospital Bamenda is a level II referral hospital in the North-West Region of Cameroon with a capacity of 400 beds and a bed occupancy rate of about 90%. The BRHL was chosen for this study for the following reasons: it is the largest public laboratory in the North-West Region with high quality and specialised services. It has a central laboratory made up of six departments and three satellite laboratories within the hospital premises. It has the capacity to run 118 different tests and a staff of 35. It serves about 170 patients (inpatients and outpatients) and about 90 clinicians (doctors, senior nurses, charge nurses and consulting nurses). It performs customer satisfaction surveys to continuously improve the services and the presence of records. Besides, the Haematology, Biochemistry and Serology services are newly accredited for competence and compliance with the ISO 15819:2012 Standard by the South African National Accreditation System.^15^

### Research design

This retrospective study reviewed secondary data collected from clinician satisfaction surveys that were carried out at the BRHL in March 2017 and November 2017. Information from the survey questionnaires and corrective action records were the primary data from which secondary data for the study were collected.

The BRHL has an advanced quality management system and conducts customer satisfaction surveys twice a year as a quality indicator for its balanced scorecard. The designed questionnaires were reviewed by the laboratory management and medical adviser^16^ and were administered to clinicians selected at random at the BRHL by the head of the customer service in March 2017 and again in November 2017 to the same group of participants. Both surveys were completed within a week during the hospital coordination meetings and the responses collected at the end of the meeting or at the offices of those who were not present in the meetings and those who were not on duty. The content of the questionnaires was explained to the participants who personally responded to the questions. The respondents were free to provide a phone number (optional) and signature (optional) so they could be contacted in case clarification was needed.

Each questionnaire was made up of rating statements and open-ended questions. The statements were assessed using a 5-point Likert ranking as follows: poor (1), fair (2), good (3), very good (4), excellent (5).^17^ The statements covered the following areas: accuracy (correctness of laboratory results), reliability (trustworthiness of laboratory results), waiting time (time between sample collection and dispatch of results), communication (management of information by the laboratory), response to emergencies (attention to emergency tests), staff-client relationship (the bond between laboratory staff and clinicians), cleanliness (neatness of the laboratory and staff), duty consciousness (awareness of laboratory staff to perform their duty promptly), specimen collection (effectiveness of phlebotomists), issuing of results (effectiveness of delivery of results), approach (the way laboratory staff attended to patients and clinicians) and general satisfaction (the overall assessment of the service). Open-ended questions included: What did the clinician like most about the laboratory? What did the clinician dislike about the laboratory, recommendations for improvements and suggestions of the tests that could be included on the laboratory test menu?

At the end of the surveys, reports were presented to laboratory staff during the staff meetings and to clinicians in the laboratory-clinic interface (hospital coordination and scientific) meeting. Action plans with corrective actions were implemented for the non-conformities (challenges) identified. Evidence of the closed gaps were maintained in action plan reports, communication logs, complaint logs and meeting notes.

#### Inclusion criteria

Data from clinicians who were part of the surveys were included in the study. This included clinicians who were present at the hospital coordination meetings and clinicians who were on duty during the weeks that the surveys were conducted. Participation was based on a random selection with verbal consent (by agreeing to respond to the questionnaire).

#### Exclusion criteria

Data from clinicians who were not part of the surveys were excluded from the study. This included clinicians who were not present at the hospital coordination meetings and clinicians who were not on duty during the weeks the surveys were conducted.

### Data collection

Data were collected by three reviewers trained to collect secondary data for the study using a structured data collection format. All available questionnaires from the two surveys were used. The data were classified by level of satisfaction as follows: responses of excellent and very good were classified as ‘satisfied’, good was classified as ‘neutral’, and fair and poor were classified as ‘dissatisfied’. Data for statements assessing: accuracy, reliability, waiting time, communication, response to emergencies, staff-client relationship, cleanliness, duty consciousness, specimen collection, issuing of results, approach and general satisfaction were decoded and entered into a Microsoft Excel 2010 spreadsheet (Microsoft Corporation, Redmond, Washington, United States). Action plans (the plan of activities in the laboratory), communication logs (records of communication with clinicians), complaint logs (records of complaints from clinicians) and meeting notes (minutes from laboratory staff meetings and laboratory-clinic interface meetings) were reviewed to capture the corrective actions implemented. Key informants (doctors, nurses, laboratory staff, laboratory management and hospital management) were contacted in case a clarification or further explanation or information was needed.

### Data analysis

Percentage frequency distributions were calculated to rate the levels of satisfaction (satisfied, neutral and dissatisfied) for each of the assessed statements. Dissatisfaction with an assessed statement was defined as a percentage score of 20% or more of dissatisfied responses. Chi-square and Fisher exact tests were used to compare the rates of satisfaction of the two surveys. A *p*-value of 0.05 or less was considered to be statistically significant.^8^

Assessed statements with 20% or higher dissatisfaction score on March 2017 were selected for review. Qualitative content analysis was conducted for the open-ended question responses by using calculated frequencies from two reviewers.^8,16,18^ Responses that were related to assessed statements with a dissatisfied score were identified as challenges. Clarification on the challenges and the corrective actions implemented were identified through a review of clinician survey reports, meeting notes, communication logs, complaint log action plans and interrogation with key informants (doctors, nurses, BRHL staff and laboratory management and hospital management).

## Results

### Customer satisfaction scores

Out of 85 questionnaires administered to the clinicians in March 2017 and 89 administered in November 2017 during the clinician satisfaction surveys, 83 and 88 clinicians responded (response rates: 97.6% for March 2017 and 98.9% for November 2017) (Table 1). Following the assessed statements in March 2017, waiting time (34.9%) registered the highest dissatisfied rate. High dissatisfaction rates were also registered for general satisfaction (26.3%), communication (24.4%), responses to emergencies (24.1%), issuing of results (22.0%), specimen collection (21.5%), approach (21.3%) and duty consciousness (21.0%). The following areas fell below the dissatisfied rate: reliability (14.5%), staff-patient relationship (11.4%) and cleanliness (7.2%) (Table 2).

**TABLE 1:** Summary of content analysis of the clinician satisfaction survey of March 2017 at the Regional Hospital Bamenda, Cameroon.

Challenges identified from open-ended questions related to the assessed statements	Number of responses
**Waiting time**	
No respect for waiting time for some emergencies, single test or grouped tests (on yellow form)	32
No explanation when waiting time is exceeded	8
No information on the waiting time for some tests	12
Shortage of staff	15
No recreational facilities	1
**Duty consciousness**	
Lateness to work	1
Ineffective presence on duty post	2
**Approach**	
Impoliteness	4
Inadequate explanation	7
**Issuing of results**	
Inadequate follow-up of pending results	10
Late issuing of results in the wards	3
**Specimen collection**	
Poor consent	2
Poor venepuncture	3
Many specimen rejections	3
Task shifting	2
**Communication**	
Inadequate explanation of procedure and difficulties	3
Lack of phone numbers of the laboratory	5
Lack of laboratory test menu	7
**Response to emergencies**	
Procedure for emergencies not respected	11
Long waiting time	19

**TABLE 2:** Distribution of responses to the assessed statements exploring clinicians’ views on laboratory services from the same population in March 2017 and November 2017 at Bamenda Regional Hospital Laboratory, Cameroon.

Statement assessed	Months	Number of participants (*N*)†	Satisfied		Neutral		Dissatisfied		*p*
			*N*	*%*	*N*	*%*	*N*	*%*	
**Accuracy**	March 2017	83	46	55.4	31	37.3	6	7.2	0.005**
	November 2017	88	64	72.7	24	27.3	0	0.0	-
**Reliability**	March 2017	83	43	51.8	28	33.7	12	14.5	< 0.001**
	November 2017	88	67	76.1	19	21.6	2	2.3	-
**Waiting time**	March 2017	83	16	19.3	38	45.8	29	34.9	0.014*
	November 2017	88	39	44.3	32	36.4	17	19.3	-
**Communication**	March 2017	82	37	45.1	25	30.5	20	24.4	0.036*
	November 2017	88	56	63.6	21	23.9	11	12.5	-
**Responses to emergencies**	March 2017	83	35	42.2	28	33.7	20	24.1	0.078
	November 2017	88	47	53.4	31	35.2	10	11.4	-
**Staff-patient relationship**	March 2017	79	36	45.6	34	43.0	9	11.4	0.014*
	November 2017	87	59	67.8	23	26.4	5	5.7	-
**Cleanliness**	March 2017	83	53	63.9	24	28.9	6	7.2	< 0.001**
	November 2017	88	76	86.4	12	13.6	0	0.0	-
**Duty consciousness**	March 2017	81	32	39.5	32	39.5	17	21.0	< 0.001*
	November 2017	86	55	64.0	27	31.4	4	4.7	-
**Specimen collection**	March 2017	79	30	38.0	32	40.5	17	21.5	0.020*
	November 2017	87	52	59.8	23	26.4	12	13.8	-
**Issuing of results**	March 2017	82	32	39.0	32	39.0	18	22.0	0.005*
	November 2017	86	53	61.6	26	30.2	7	8.1	-
**Approach**	March 2017	80	30	37.5	33	41.3	17	21.3	0.007*
	November 2017	83	50	60.2	26	31.3	7	8.4	-
**General satisfaction**	March 2017	80	25	31.2	34	42.5	21	26.3	< 0.001*
	November 2017	83	51	61.4	24	28.9	8	9.6	-

* , Significance at ≤ 0.05 for chi-square test;

** , Significance at ≤ 0.05 for Fisher’s exact test.

† , Data from participants who failed to answer any of the questions in the assessed statements were excluded from analyses of that particular question.

In November 2017, no assessed statements had a dissatisfied score. Between March 2017 and November 2017, statistically significant reductions in dissatisfied scores were registered for: waiting time (34.9% to 19.3%, *p* = 0.014), general satisfaction (26.3% to 9.6%, *p* < 0.001), communication (24.4% to 12.5%, *p* = 0.036), issuing of results (22.0% to 8.1%, *p* = 0.005), specimen collection (21.5% to 13.8%, *p* = 0.020), approach (21.3% to 8.4%, *p* = 0.007), and duty consciousness (21.0% to 4.7%, *p* < 0.001). Although not statistically significant, there was a general decrease in dissatisfied scores between the two surveys for responses to emergencies, reliability and the staff-patient relationship. In addition, accuracy and cleanliness had reduced dissatisfied scores on the November 2017 survey, although they were not considered for evaluation, since they did not register dissatisfied rates below 20% on the March 2017 survey.

### Open-ended responses

**Waiting time:** Waiting time received the highest number of open-ended responses (Table 2). Respondents cited the lack of respect for waiting time for some emergencies, single or grouped tests, no explanation when the waiting time was delayed, inadequate information about the laboratory services, shortage of staff and lack of relaxation facilities such as a television set, video or audio player. Some clinicians did not use the Stat Request Forms (Lab-Rap-Form) or indicate the emergency code ‘URGENT!’ on request forms in cases of emergency. Thus, their requests were treated as routine. For corrective actions, clinicians were oriented on the various processes and procedures in the laboratory, the waiting time for some tests was re-established, a test menu with waiting times was redistributed and posted in all wards, issuing of results at the top of each hour was intensified, laboratory staff were re-trained on good customer service practice, more staff were hired and a staff retention policy proposed to the hospital management. Additionally, a proposal for the purchase of a television and a sound system was made to the hospital management.

**Duty consciousness:** Concerning duty consciousness, clinicians commented on lateness to work and ineffective presence at the duty post. Although the hospital had a biometric time clock system that was used to monitor staff attendance, the details were evaluated at the end of the month. Thus, the effective presence of staff on duty was not promptly monitored using the biometric system. For corrective actions, the laboratory staff were cautioned about having a poor attitude and an attendance register was introduced in the laboratory by the laboratory management.

**Approach**: For approach, clinicians identified as challenges the lack of politeness of laboratory staff and inadequate explanations to patients or clinicians. For corrective actions, the laboratory staff were cautioned about having a poor attitude and re-trained on the importance of good customer service.

**Issuing of results:** Clinicians identified inadequate follow-up on pending results and delay in the dispatch of some results, especially for patients admitted in the wards, as the main challenges. For corrective actions, the laboratory staff were cautioned about having a poor attitude, the laboratory management regularly made supervision tours around the wards to monitor the activities of the laboratory, and the results dispatch registers were monitored daily.

**Specimen collection:** For specimen collection, clinicians reported poor patient consent, poor venepuncture, many specimen rejections and task shifting (laboratory phlebotomist to nurses for patients admitted in the wards) as challenges. For corrective actions, the laboratory staff and clinicians were educated and trained on the specimen collection procedures and the need for proper specimen collection.

**Communication:** For communication, clinicians identified the following challenges: an inadequate explanation of procedures or difficulties and unavailability of laboratory information (phone numbers of some of the laboratory managers and test menu) to some clinicians. For corrective actions, adequate information on the laboratory services was provided and the hospital intercommunication system was maintained.

**Response to emergencies:** Clinicians identified the following challenges related to response to emergencies: lack of respect for the procedures and waiting time for emergency requests. For corrective actions, laboratory staff were cautioned about having a poor attitude towards work, clinicians were oriented on the procedure for handling emergency requests, emergency requests were tracked to monitor waiting times and laboratory staff were cautioned about the need for adequate communication.

## Discussion

The purpose of this study was to assess clinicians’ satisfaction, identify what challenges clinicians faced when accessing laboratory services, identify corrective actions implemented in response to those challenges and document changes in satisfaction. A good response rate^16^ was registered for both surveys, which was attributed to the fact that most of the questionnaires were issued to the respondents (clinicians) during hospital coordination meetings, where almost all of the respondents were present and the responses were collected at the end of the meeting. For both surveys, some of the questionnaires administered to clinicians at their offices were not returned. Respondents knew the surveys were routine and that they were an avenue for them to give their feedback, express their challenges and make proposals for the improvement of the laboratory services.^8^

The dissatisfaction rate in relation to waiting time in the March 2017 survey was the highest. Although the laboratory had an advanced quality management system in place for the management of patients, the system was not respected by the laboratory staff. In addition, the absence of the hospital intercommunication system affected the flow of information from the laboratory to the wards and clinicians. Due to the implementation of some of the corrective actions, there was a significant reduction in the dissatisfaction rate concerning waiting time on the November 2017 survey. The clinicians (especially those newly hired) created greater awareness of the activities and relationships between clinicians and the laboratory. They understood why the waiting time of some tests were longer than others and could, in turn, inform their patients. Furthermore, the maintenance of the hospital intercommunication system strengthened communication between the laboratory and clinicians.

Similar dissatisfaction rates have also been identified in many other studies. Oja et al., reported a dissatisfactory score for waiting time by clinicians in a study in Finland.^8^ They attributed this to inadequate information about the waiting time of emergency requests, lack of respect for procedures for emergency requests and high prices for emergency tests.^8^ For corrective action, the laboratory informed the clinicians about the waiting time and advised them to order emergency tests for emergency cases.^8^ Steindel and Howanitz in a survey of clinicians in the United States also reported low satisfaction rates for waiting time.^7^ They observed that the waiting time for laboratory services was too long and the clinicians did not consider the laboratory sensitive enough to handle their emergency requests.^7^ Allen and Harris^19^ and Boyde et al.,^20^ in the United Kingdom also reported dissatisfactory rates for waiting time by clinicians. Teklemariam et al. reported a satisfactory score for the waiting time for notification of critical values and timely results for HIV testing, but this was for a single test,^9^ whereas the report from our study was for the entire laboratory. Zarbo et al. and Zarbo, in another study in the United States, reported low satisfaction scores by clinicians that were related to timeliness of reporting,^11, 21^ and although corrective actions were implemented in the study in Finland, there was no significant change in the dissatisfaction rate.

Duty consciousness received a high dissatisfaction rate score on the March 2017 survey. The corrective actions and the introduction of the laboratory attendance register by the laboratory management significantly reduced the dissatisfaction rate. This improved supervision of staff by the laboratory management, because details of staff attendance and movements, including when they went on break, were monitored promptly and daily. Oja et al. in Finland, observed a low dissatisfaction rate for the attitude that was not appropriate for comparison during their statistical analysis.^8^

The approach by the laboratory staff also had a high dissatisfaction rate score in March 2017, this was attributed to a poor attitude on the part of some laboratory staff, possibly due to burnout from high workload. The corrective actions implemented significantly reduced the dissatisfaction rate in November 2017. The re-training and re-orientation of laboratory staff and the hiring of more workers also reduced burnout. Other studies did not assess the approach.^7,8,11^

Clinicians were also dissatisfied with the issuing of results on the March 2017 survey. The laboratory staff were cautioned on attitude, and the corrective actions implemented significantly reduced the dissatisfaction rate. Oja et al., in Finland, reported a non-significant rate of dissatisfaction for missing results^8^ and Zarbo^21^ reported a dissatisfaction rate for notification of abnormal results.

Specimen collection registered a high dissatisfaction rate on the March 2017 survey. According to key informants, this was attributed to inadequate training and orientation and failure to respect laboratory policies and procedures. Many laboratory staff and clinicians were newly hired and were not trained on specimen collection. The corrective actions implemented significantly reduced the dissatisfaction rate. The clinicians became aware that they have to assist in specimen collection in emergency cases, including difficult cases, during routine working hours (7:30 to 15:30 pm). Oja et al., in Finland, reported a significant reduction in dissatisfaction rate for phlebotomist rounds, after the rounds were rescheduled in cooperation with the in-patient unit and other clinical units adjusted their work pattern to integrate better with the laboratory service.^8^

Communication registered a high rate of dissatisfaction among the assessed statements in the March 2017 survey. According to key informants, this was attributed to the fact that some of the wards were recently renovated and information posted on the walls had been removed. Some of the clinicians were newly hired or recruited and had not received an adequate orientation on the laboratory procedures and policies. Although the main communication tools of the laboratory were in place, the laboratory did not adequately use them and verbal communication was still a problem. The laboratory did not register some of the verbal complaints presented by the clinicians for follow-up, and the complaints were persistent. Information from key informants identified poor verbal communication of laboratory staff as the main cause of most of the challenges, which included long waiting time, many specimen rejections and poor follow-up of pending results. Normally, after specimen collection in the wards, the phlebotomist was supposed to inform the patient of the exact hour of the day to collect results, but some phlebotomists informed them of the duration (for example, after 2 hours). This caused some of the patients to speculate the exact hour of the day to collect their results and resulted in numerous complains about waiting time even when the time had not been exceeded. Besides, the laboratory results of patients admitted to the wards were dispatched directly to the clinicians, but some patients and caregivers presented to the laboratory separately to collect results that had already been issued to the wards. This was due to the failure to provide adequate information to the patient or caregiver during specimen collection by the laboratory. The corrective actions implemented significantly reduced the dissatisfaction rate of communication in the November 2017 survey. Clinicians could better orient their patients on the functioning of the laboratory. Oja et al.,^8,^ in Finland reported a low dissatisfaction rate in their study that was not appropriate for comparison during statistical analysis.

Response to emergencies registered a high dissatisfaction rate in the March 2017 survey. Key informants attributed this to the poor attitude of the staff and lack of adequate information on laboratory services provided to clinicians. The corrective actions implemented significantly reduced the dissatisfaction rate. Oja et al., in Finland, observed a non-significant reduction in dissatisfaction rate for scheduling of phlebotomists during emergency hours.

General satisfaction was the general impression of the clinicians about the laboratory services. General satisfaction registered a high rate of dissatisfaction among the assessed statements on the March 2017 survey. This was due to the accumulation of challenges from all the other assessed statements. The corrective actions implemented following the identification of all the challenges accounted for the significant reduction in the dissatisfaction rate in the November 2017 survey. Several other studies did not consider the general satisfaction with laboratory services.^7,8,11^

### Recommendations

A significant increase in customer (clinicians) satisfaction level of the BRHL can be achieved through cooperation among hospital management, laboratory management, clinicians and other hospital staff. It is recommended that: the government should improve laboratory infrastructure to boost patient flow, employ more staff to replace those who transfer or retire and provide more automated machines to increase efficiency and reduce waiting times. The hospital management should: provide an interface system to link laboratory equipment to the laboratory information system to reduce clerical errors, maintain the internal communication system in the hospital, provide a web-based communication system that links the laboratory service to patients and clinicians, notify them when their results are ready, remind them of their appointments with the laboratory and allow logging of issues with laboratory services, provide equipment such as a television set, video or audio player to entertain customers, especially when they are waiting to be served, hire more staff to handle the workload, sign engagements with hired workers to guarantee their job security and motivation, and retain existing staff to maintain continuity, train other hospital staff on the importance of customer satisfaction, support the laboratory in the effective implementation of corrective actions, and plant close-circuit television cameras in the departments of the laboratory and hospitals to ease supervision and monitoring of staff activities and customers.

Clinicians should respect the policies and procedures of the laboratory and hospital. They should follow their appointments and report any incidents to the laboratory management, educate and counsel their patients before sending them to the laboratory, communicate well with their patients and the laboratory management in case of any incident, and receive and direct patients properly to avoid cross-aggregation with other services. The laboratory management and staff should positively change their attitude, respect all laboratory policies and procedures, intensify supervision, regularly train new and old staff, effectively implement corrective and preventive actions in real-time, intensify meetings (laboratory-clinic interface) with the users of the laboratory, educate the public on the use of laboratory services by making more frequent public talks over the radio and television, and revise the questionnaire to include more open-ended questions.

### Limitations

Since this is a retrospective study, we could not control the consistency of the data collected. Our study was based on the data collected, so we were limited to the assessed statements or questions. The fact that some participants provided their phone numbers might not be a bias since they knew that they could be called up as key informants and this was optional.

### Conclusion

According to our study, waiting time was a major cause of clinician dissatisfaction with laboratory services. To maintain reduced dissatisfaction rates in clinical laboratories, it is important to continuously identify challenges and effectively implement corrective actions.

## References

[1] Kaye AD, Okanlawon OJ, Urman RD Clinical performance feedback and quality improvement opportunities for perioperative physicians. Adv Med Educ Pract. 2014;5:115 10.2147/AMEP.S62165 24833948 PMC4014376

[2] Veloski J, Boex JR, Grasberger MJ, et al Systematic review of the literature on assessment, feedback and physicians’ clinical performance: BEME Guide No. 7. Med Teach. 2006;28(2):117–128. 10.1080/01421590600622665 16707292

[3] Mugford M, Banfield P, O’Hanlon M Effects of feedback of information on clinical practice: A review. BMJ. 1991;303(6799):398–402. 10.1136/bmj.303.6799.398 1912809 PMC1670701

[4] ISO E., 15189 Medical laboratories – Particular requirements for quality and competence. Geneva: International Organization for Standardization.

[5] College of American Pathologists Laboratory general checklist: Laboratory accreditation program. Northfeld, IL: College of American Pathologists, 2005.

[6] ISO, I ISO/IEC17025: 2005 General requirements for the competence of testing and calibration laboratories. ISO, Geneva; 2005.

[7] Steindel SJ, Howanitz PJ Physician satisfaction and emergency department laboratory test turnaround time: Observations based on College of American Pathologists Q-Probes studies. Arch Pathol Lab Med. 2001;125(7):863–871. 11419969 10.5858/2001-125-0863-PSAEDL

[8] Oja PI, Kouri TT, Pakarinen AJ From customer satisfaction survey to corrective actions in laboratory services in a university hospital. Int J Qual Health Care. 2006;18(6):422–428. 10.1093/intqhc/mzl050 17003077

[9] Teklemariam Z, Mekonnen A, Kedir H, et al Clients and clinician satisfaction with laboratory services at selected government hospitals in eastern Ethiopia. BMC Res Notes. 2013;6(1):15 10.1186/1756-0500-6-15 23324260 PMC3556148

[10] Jones BJ, Bekeris LG, Nakhleh RF, et al Physician satisfaction with clinical laboratory services: A College of American Pathologists Q-probes study of 138 institutions. Arch Pathol Lab Med. 2009;133(1):38–43. 19123734 10.5858/133.1.38

[11] Zarbo RJ, Nakhleh RE, Walsh M, et al Customer satisfaction in anatomic pathology: A College of American Pathologists Q-Probes study of 3065 physician surveys from 94 laboratories. Arch Pathol Lab Med. 2003;127(1):23–29. 12521362 10.5858/2003-127-23-CSIA

[12] Yao K, McKinney B, Murphy A, et al Improving quality management systems of laboratories in developing countries: An innovative training approach to accelerate laboratory accreditation. Am J Clin Pathol. 2010;134(3): 401–409. 10.1309/AJCPNBBL53FWUIQJ 20716796

[13] Yao K, Maruta T, Luman ET, et al The SLMTA programme: Transforming the laboratory landscape in developing countries. Afr J Lab Med. 2014;3(2), Art. #194, 8 pages. 10.4102/ajlm.v3i2.194 PMC4703335 26752335

[14] Ndasi J, Dimite L, Mbome V., et al Decentralised facility-based training as an alternative model for SLMTA implementation: The Cameroon experience. Afr J Lab Med. 2014 3(2), Art. #231, 6 pages. 10.4102/ajlm.v3i2.231 PMC5637810 29043194

[15] SANAS [cited 2020 Jan 08]. Available from: https://www.sanas.co.za/Pages/index.aspx

[16] Kelley K, Clark B, Brown N, et al Good practice in the conduct and reporting of survey research. Int J Qual Health Care. 2003;15(3):261–266. 10.1093/intqhc/mzg031 12803354

[17] Streiner DL, Norman GR, Cairney J Health measurement scales: A practical guide to their development and use. Oxford University Press, Oxford; 2015.

[18] Morse JM, Field PA Nursing research: The application of qualitative approaches. Nelson Thornes Ltd, Cheltenham; 1995.

[19] Allen K, Harris C Measure of satisfaction of general practitioners with the chemical pathology services in Leeds Western Health District. Ann Clin Biochem. 1992;29(3):331–336. 10.1177/000456329202900314 1610108

[20] Boyde AM, Earl R, Fardell S, et al Lessons for the laboratory from a general practitioner survey. J Clin Pathol. 1997;50(4):283–287. 10.1136/jcp.50.4.283 9215142 PMC499876

[21] Zarbo RJ Determining customer satisfaction in anatomic pathology. Arch Pathol Lab Med. 2006;130(5):645–649. 16683881 10.5858/2006-130-645-DCSIAP

